# Influence of Power Delivery Timing on the Energetics and Biomechanics of Humans Wearing a Hip Exoskeleton

**DOI:** 10.3389/fbioe.2017.00004

**Published:** 2017-03-08

**Authors:** Aaron J. Young, Jessica Foss, Hannah Gannon, Daniel P. Ferris

**Affiliations:** ^1^Woodruff School of Mechanical Engineering, Georgia Institute of Technology, Atlanta, GA, USA; ^2^Department of Biomedical Engineering, University of Michigan, Ann Arbor, MI, USA; ^3^School of Kinesiology, University of Michigan, Ann Arbor, MI, USA

**Keywords:** powered orthosis, robotic control, metabolic cost, biomechanics, human walking, hip exoskeleton

## Abstract

A broad goal in the field of powered lower limb exoskeletons is to reduce the metabolic cost of walking. Ankle exoskeletons have successfully achieved this goal by correctly timing a plantarflexor torque during late stance phase. Hip exoskeletons have the potential to assist with both flexion and extension during walking gait, but the optimal timing for maximally reducing metabolic cost is unknown. The focus of our study was to determine the best assistance timing for applying hip assistance through a pneumatic exoskeleton on human subjects. Ten non-impaired subjects walked with a powered hip exoskeleton, and both hip flexion and extension assistance were separately provided at different actuation timings using a simple burst controller. The largest average across-subject reduction in metabolic cost for hip extension was at 90% of the gait cycle (just prior to heel contact) and for hip flexion was at 50% of the gait cycle; this resulted in an 8.4 and 6.1% metabolic reduction, respectively, compared to walking with the unpowered exoskeleton. However, the ideal timing for both flexion and extension assistance varied across subjects. When selecting the assistance timing that maximally reduced metabolic cost for each subject, average metabolic cost for hip extension was 10.3% lower and hip flexion was 9.7% lower than the unpowered condition. When taking into account user preference, we found that subject preference did not correlate with metabolic cost. This indicated that user feedback was a poor method of determining the most metabolically efficient assistance power timing. The findings of this study are relevant to developers of exoskeletons that have a powered hip component to assist during human walking gait.

## Introduction

A number of research and industry groups are developing powered lower limb exoskeletons to help people in industry (Hodson, 2014; Lamothe, 2014), military (Zoss et al., 2006; Gregorczyk et al., 2010; Raytheon XOS 2 Exoskeleton, Second-Generation Robotics Suit: Army-Technology, 2010; France’s Slender Hercule Exoskeleton Is No Lightweight, 2012; Asbeck et al., 2015), and healthcare settings (Gancet et al., 2012; Zeilig et al., 2012; Kolakowsky-Hayner et al., 2013; Sczesny-Kaiser et al., 2013; Farris et al., 2014). Progress in hardware development has been rapid and widespread (Huo et al., 2014), but control over these advanced devices still needs significant improvement for exoskeletons to be adopted widely in everyday use (Yan et al., 2015). Hip and ankle muscles are the largest contributors for providing the necessary mechanical power to sustain human walking (Sawicki and Ferris, 2009; Umberger and Rubenson, 2011), and a large number of previous studies have considered how to optimize ankle exoskeletons and controllers (Ferris et al., 2005, 2006; Gordon et al., 2006; Cain et al., 2007; Gordon and Ferris, 2007; Norris et al., 2007; Sawicki and Ferris, 2008, 2009; Malcolm et al., 2009; Koller et al., 2015). Recent work has shown that a variety of different ankle exoskeletons can effectively reduce the energetic cost of walking by providing plantarflexor power at the proper time point in the gait cycle (Malcolm et al., 2013; Mooney et al., 2014; Collins et al., 2015). With the goal of making similar advances at the hip, our work focuses on a hip exoskeleton and testing its efficacy of torque delivery at the hip for both flexion and extension assistance. Several research groups have begun recently developing hip exoskeletons both for able-bodied assistance (Lewis and Ferris, 2011; Lenzi et al., 2013; Giovacchini et al., 2014) and for disabled populations (Arazpour et al., 2012, 2014; Buesing et al., 2015). Our study is relevant to researchers developing walking controllers for exoskeletons that include a powered hip joint or even passive designs that have targeted storage and return of energy at the hip. There is a clear need for researchers to study the biomechanics of lower limb exoskeletons to develop the most efficacious strategies of controlling exoskeletons to aid in human walking for both augmenting human performance and assisting the disabled (Ferris, 2009).

Recent work with hip and ankle exoskeletons has focused on the goal of reducing the metabolic cost of walking, and researchers have succeeded in a number of experiments (Ronsse et al., 2011; Mooney et al., 2014; Ding et al., 2016a,b; Ruiz Garate et al., 2016; Seo et al., 2016). Research groups have used both rigid exoskeletons (Ruiz Garate et al., 2016), as we use in our experiment, and soft exoskeletons to test the effect of different hip assistance strategies on metabolic output (Ding et al., 2016a; Panizzolo et al., 2016). A wide variety of control systems have been proposed (Aguirre-Ollinger, 2013; Jang et al., 2015; Koller et al., 2015; Oh et al., 2015; Takahashi et al., 2015; Wu et al., 2015; Yan et al., 2015; Ao et al., 2016; Chen et al., 2016; Ding et al., 2016b; Zhang et al., 2016), but typically tests are done on a unique device with only one controller. Oscillator-based controllers tend to be popular for hip exoskeletons due to the reliance on only hip joint angle sensing for control (Ronsse et al., 2011; Giovacchini et al., 2014; Seo et al., 2016; Yan et al., 2016; Sugar et al., 2017). More recent exoskeletons are becoming completely autonomous, using oscillation-based approaches to provide walking assistance that reduces the metabolic cost of walking (Seo et al., 2016).

The overall aim of this work was to determine the optimal timing for supplying hip power using an exoskeleton to assist human walking. This is a useful goal to aid the field in understanding the relationship between hip assistance timing and metabolic cost of walking. The experiment aimed to identify separately the value of hip flexion and hip extension assistance and determine the best timing interval during the gait cycle for supplying powered assistance for both movement types. The primary outcome measure was the metabolic cost of walking. We hypothesized that the controller timings that generated a pattern of exoskeleton torque most similar to the biological torque pattern would produce the largest reduction in metabolic cost (Malcolm et al., 2013). A secondary outcome measure was user preference. We tested the hypothesis that users would prefer conditions with powered assistive timing that incurred a lower metabolic cost on the user. The primary aim achieved was assessing the effects of hip timing on human performance using an exoskeleton.

## Materials and Methods

### Experimental Protocol

**Figure 1:**
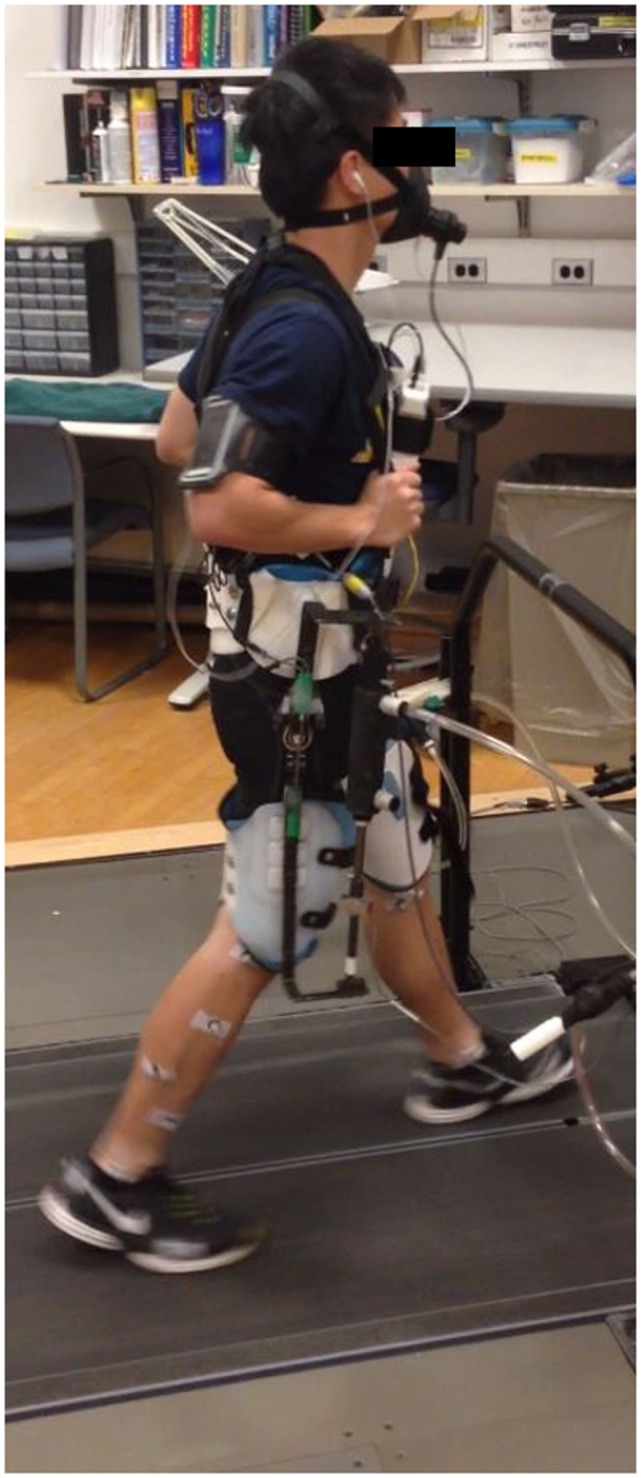
**An experimental setup of one subject wearing our exoskeleton**.

The following study protocol was approved by the University of Michigan Institutional Review Board. Ten able-bodied subjects (six females and four males) gave written consent and completed the following protocol. The subject number (*n* = 10) was based on a power analysis of metabolic cost from prior related work (Young et al., 2015) comparing metabolic cost of two different controllers on the same exoskeleton hardware with type I error set to 0.05 and type II error set to 0.2. Subjects had an average age of 20.9 years (SD, 3.8), height of 1.74 m (SD, 0.10 m), and weight of 68.2 kg (SD, 13.9). Each subject received training using the hip exoskeleton on a day prior to the experiment that included walking with both flexion and extension assistance to ensure that they were comfortable and accommodated to the exoskeleton. On the day of the experiment, each subject began with a 10-minute experimental training session. The subjects were trained to walk in the exoskeleton, starting at lower speeds and powers and increasing to full speed and power for both flexion and extension conditions. Each subject wore a custom-built hip exoskeleton (see Figure 1) that we designed. Pneumatic piston actuators (BIMBA) were attached above and below the hip in linkages attached to the exoskeleton to provide hip flexion and hip extension assistance. Load cells were placed in series with the actuators to measure the force provided by the exoskeleton on the user’s body. Subjects were individually fitted to the exoskeleton with a waist band, shoulder straps, and adjustable thigh cuffs. A full lower limb reflective marker set was placed on the human subject before they donned the exoskeleton to measure their lower limb movement profile.

Metabolic energy expenditure was measured using a system for indirect calorimetry (Oxycon Mobile, CareFusion). Before subjects began walking, we measured their resting metabolic rate for 3 min while they stood still in the exoskeleton. Subjects walked at 1.15 m/s on a split-belt instrumented treadmill (Bertec’s Fully Instrumented Split-Belt Treadmill) that measured their ground reaction forces on both the right and left sides. During the experimental training session, subjects selected a comfortable walking cadence based on an audible metronome played through a headset so that they could listen and walk at the same cadence throughout the entire experiment. The self-selected cadence was used to determine the actuation timing based on a percentage of the subjects’ gait cycle. This was determined by detecting heel contact with the instrumented force treadmill and using the step duration (based on self-selected cadence) to determine the percent gait cycle continuously in real time. This actuation timing determined when torque would be supplied to the exoskeleton.

Subjects walked for 10 min at a time in nine different experimental assistance timing conditions. Each condition provided exoskeleton assistance at different times during the gait cycle. A real-time control system (Control Desk, dSpace, Inc.) coordinated exoskeleton onset and offset timing signals. The onset timing for each leg was calibrated to heel strike (0% of gait cycle) based on each subject’s self-selected cadence. Four powered hip extension conditions were tested corresponding with onsets at 80, 90 (prior to heel strike), 0 (heel strike), and 10% (after heel strike). Four powered hip flexion conditions were tested corresponding with onsets at 30, 40, 50, and 60% of the gait cycle. The offset timing of the actuators was always 25% of the gait cycle after the onset, which is based on the timing window duration that maximized assistance in a previous study (Malcolm et al., 2013). The control system was such that the valves were opened fully during the duration of the power delivery window and closed at the offset point. This created a nearly constant pneumatic pressure in the system during the delivery window. Thus, the system acts like a burst controller and provides a burst of energy during the delivery window that is constant across the different conditions. The ninth condition was walking with the exoskeleton unpowered. The order of the nine conditions was randomized, and subjects were allowed optional breaks between each 10-min condition. Mandatory breaks were taken after every 30 min of walking.

During each condition, subjects were asked to identify whether they preferred the current condition compared to the previous condition. Subjects answered in a yes or no format by a head nod while walking. This subject feedback was recorded between all consecutive conditions, which varied based on subject due to experimental randomization.

### Metabolic Analysis

The primary outcome measure for this experiment was the energetic cost of walking with the exoskeleton. We estimated metabolic energy expenditure based on the formula from (Brockway 1987). For each condition, metabolic data from the last 3 min of each 10-min trial were used to determine metabolic energy expenditure. The metabolic cost of walking was determined by subtracting the metabolic energy expenditure of the subject while standing still from each walking measurement. We calculated the metabolic cost of walking for each of the eight powered conditions and the unpowered condition. We also analyzed the effect of choosing the best assistance time for reducing metabolic cost in the gait cycle on a subject-by-subject basis by creating an “optimized” condition. This consisted only of each subject’s assistance timing that minimized metabolic cost. This was calculated separately for flexion and extension assistance.

### Biomechanical Analysis

Biomechanical analysis was performed to determine changes between conditions for the hip, knee, and ankle joint kinematics and kinetics. We combined motion capture data from a 10 camera Vicon system with 6-DOF force plate information for each leg from the treadmill force plates as inputs into Visual 3D (C-Motion). Using Visual 3D, a lower limb model was created to determine joint angles, moments, and powers using inverse dynamics. We also calculated the torques generated by the exoskeleton onto the user based on the load cell signal from each leg and the moment arm. Exoskeleton hip power was calculated based on the calculated torque and the exoskeleton hip velocity. We excluded 3 of the 10 subjects for biomechanical analysis due to the exoskeleton blocking their markers during the walking trials such that motion tracking was impossible.

### Statistical Analysis

A repeated measures one-way ANOVA statistical model was used to compare differences in metabolic cost of walking across different timing conditions. A Bonferroni *post hoc* test was used to create pairwise comparisons between the different conditions and correct for multiple measures by setting the global type I error to 0.05. A regression analysis was run between user preference and metabolic cost.

## Results

### Metabolic Results

**Figure 2:**
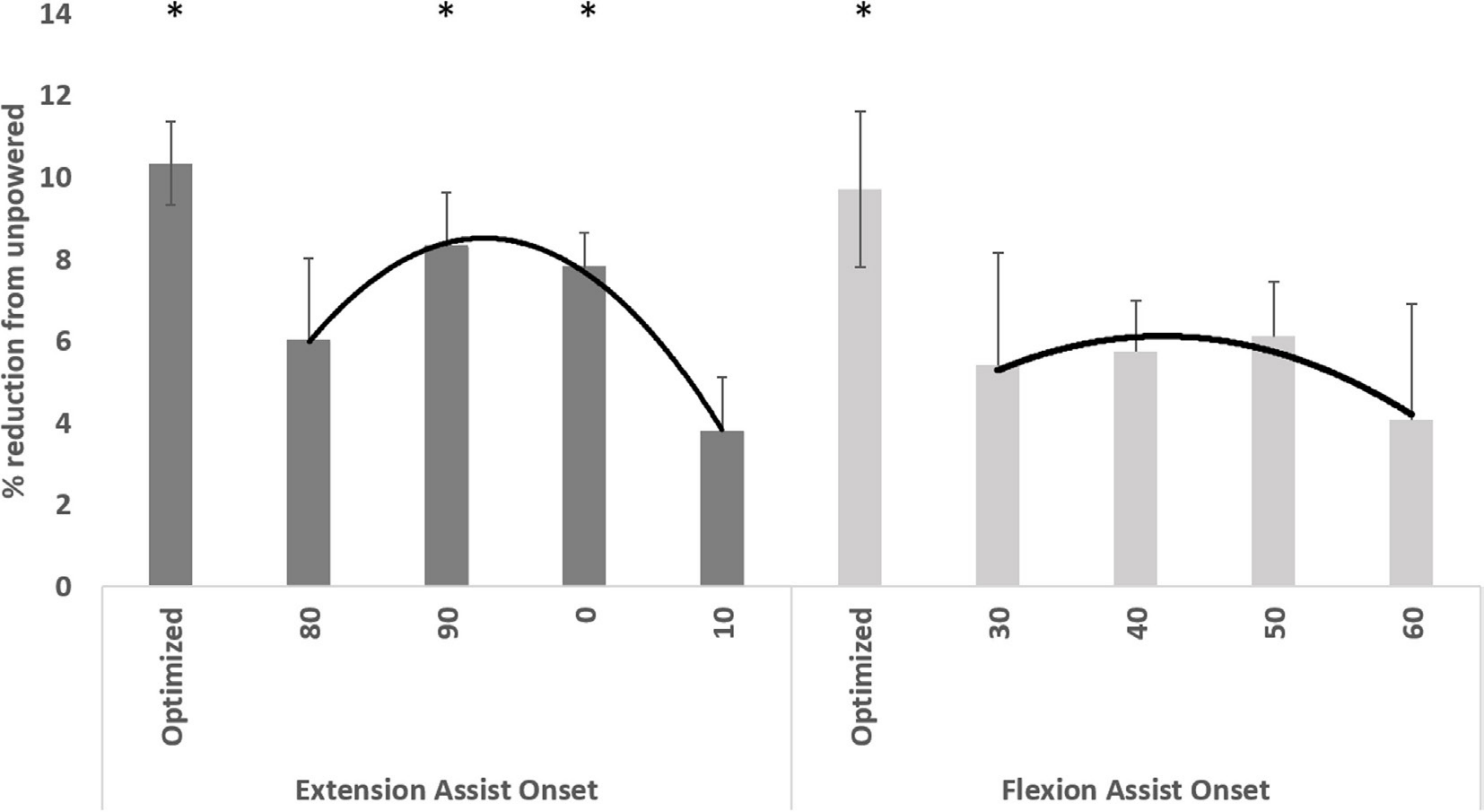
**Metabolic cost of different powered conditions relative to the unpowered condition**. The “optimized” condition for both hip flexion and extension was calculated by only using the best assistance timing (the timing that had the lowest metabolic cost) on a subject-by-subject basis. In all conditions, metabolic cost was lower on average compared to the unpowered condition. Stars indicate conditions that had significantly (*p* < 0.05) lower metabolic cost compared to the unpowered condition in the pairwise Bonferroni correction *post hoc* tests. Data are averaged across the 10 users, and error bars show ±1 SEM.

The average metabolic cost of walking across subjects was reduced in all powered conditions, regardless of activation timing, compared to the unpowered condition (Figure 2). The overall ANOVA results found that timing was a significant factor (*p* < 0.05). *Post hoc* testing revealed that hip extension onset at both 90 and 0% of the gait cycle resulted in a significant decrease in metabolic cost compared to the unpowered condition (*p* < 0.05). On average across users, the best timing for hip extension assistance occurred with an onset at 90% of the gait cycle and at 50% of the gait cycle for hip flexion assistance. However, not all subjects had the same assistance timing for both hip flexion and extension assistance that maximally reduced metabolic cost (see Table 1). We created an “optimized” condition for both hip flexion and extension assistance by selecting the assistance timing condition with a maximum reduction in metabolic cost and then averaging all of the values. The optimized condition (Figure 2) for hip flexion and extension reduced metabolic cost by 9.7 and 10.3%, respectively, compared to the unpowered condition (*p* < 0.05 for both).

**Table 1:** **Metabolic cost of walking for each subject for all conditions, normalized to body weight (W/kg)**.

Subject	Standing	Unpowered	Extension assist onset				Flexion assist onset			
			80	90	0	10	30	40	50	60
1	1.12	3.62	3.43	3.52	**3.36**	3.71	3.07	3.69	**3.03**	3.20
2	1.63	4.35	4.25	**3.81**	3.99	4.14	3.87	3.86	4.08	**3.76**
3	1.82	3.95	3.81	**3.63**	3.68	3.69	4.10	**3.84**	3.87	3.86
4	1.36	3.91	3.73	3.58	3.60	**3.51**	4.16	**3.64**	3.84	4.25
5	1.29	5.00	**4.27**	4.40	4.72	4.81	**3.91**	4.83	4.56	4.02
6	1.21	4.18	**3.93**	4.03	4.01	4.14	3.98	**3.72**	4.00	4.03
7	1.73	4.35	**3.73**	3.89	3.75	4.05	4.27	**4.15**	4.18	4.33
8	1.48	3.56	3.81	3.44	**3.25**	3.50	3.56	**3.33**	**3.33**	3.70
9	1.45	3.85	3.56	**3.53**	3.57	3.91	3.76	3.70	**3.67**	4.03
10	1.79	4.81	4.34	**4.14**	4.37	4.47	4.45	**4.38**	4.48	4.45

*The minimum metabolic output for both extension and flexion assistance is set in bold*.

We found a minimum for hip extension assistance at 92.2% of the gait cycle using a parabolic fit (second-order polynomial) to the metabolic data. This corresponded to a hypothetical maximum metabolic reduction of 8.8% compared to the unpowered condition. This represented 85% of the gain compared to the optimized condition for hip extension and thus is likely sufficient for generalized use across subjects (though not optimal). The polynomial fit (see Figure 2 for graphical representation) had an R^2^ value of.99 for the hip extension data indicating a good fit. The minimum of the parabolic fit for hip flexion assistance occurred at the onset point of 40.2% of the gait cycle and had a hypothetical maximum metabolic reduction of 6.2%. This represented 64% of the gain compared to the optimized condition for hip flexion. This indicated that some subject-specific tuning could be useful. However, the data for the 10 subjects indicate that 8 of 10 subjects had their lowest metabolic costs using hip flexion assistance at either 40 or 50% onset, and likely a timing onset in this range of the gait cycle is suitable. The polynomial fit (see Figure 2 for graphical representation) had an R^2^ value of 0.87 for the hip flexion data indicating a decent fit, but not as good as the extension fit.

### Subject Preferences

**Figure 3:**
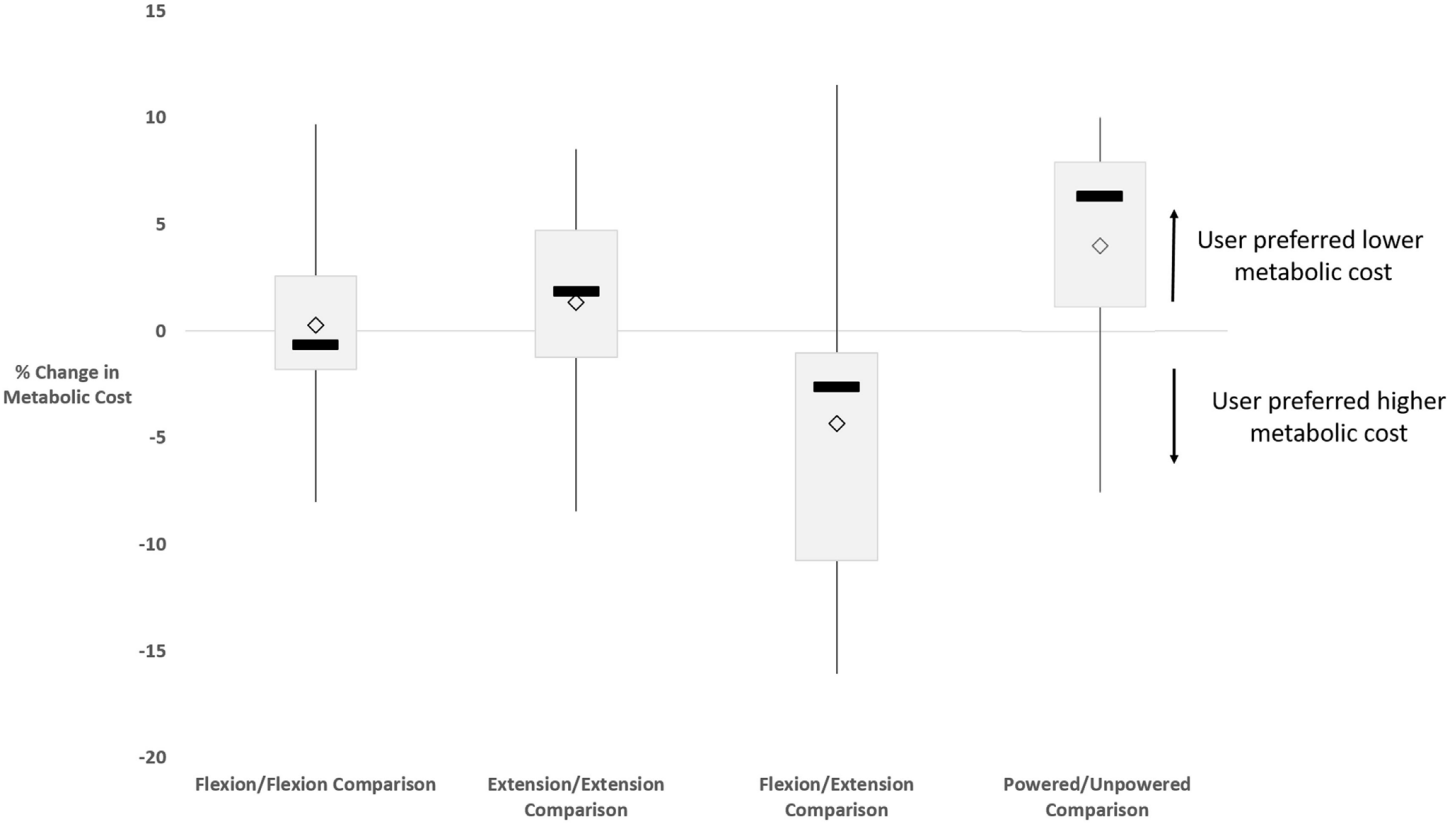
**Box and whiskers plot showing user preference versus change in metabolic cost of walking between conditions**. The black bars show the data median, and the diamonds show the data mean. The box indicates the range of the second and third data quartiles. The bars show the range of the first quartile (lower) and fourth quartile (upper). A negative change corresponded to a user selecting a condition that incurred an increased metabolic cost, while a positive change corresponded to a user selecting a condition that incurred a reduced metabolic cost. Distributions are shown for user preference comparisons (from left to right) between flexion conditions, between extension conditions, across flexion and extension conditions, and across powered and unpowered conditions. The compared conditions vary from subject to subject based on the randomized condition order, as user preference comparisons were made between consecutive conditions. From this figure, our results indicate that subject preference is a poor way to try to tune the assistance timing parameter for reducing metabolic cost.

Subject preference did not correspond to the reduction of the metabolic cost of walking (*p* = 0.752 based on regression). When subjects were asked to compare between similar walking conditions during the experiment (one flexion condition to another flexion condition or one extension condition to another extension condition), their preferences did not correlate with a reduction of metabolic cost (Figure 3). We asked subjects their preference of conditions during the experiment rather than afterward to ensure that there was no confusion between any of the nine walking conditions. When comparing between flexion and extension conditions, subjects tended to prefer hip flexion assistance regardless of the change (upward or downward) in metabolic cost between the two conditions. Nine of the 10 subjects preferred hip flexion assistance over hip extension assistance, but hip extension assistance tended to reduce a subjects’ metabolic cost more than hip flexion assistance. Thus, subjects tended to prefer conditions that had a higher metabolic cost when comparing hip flexion and hip extension assistance. In the case of comparing between powered and unpowered conditions, subjects tended to prefer powered conditions that typically had the lower metabolic cost.

### Controller Analysis

**Figure 4:**
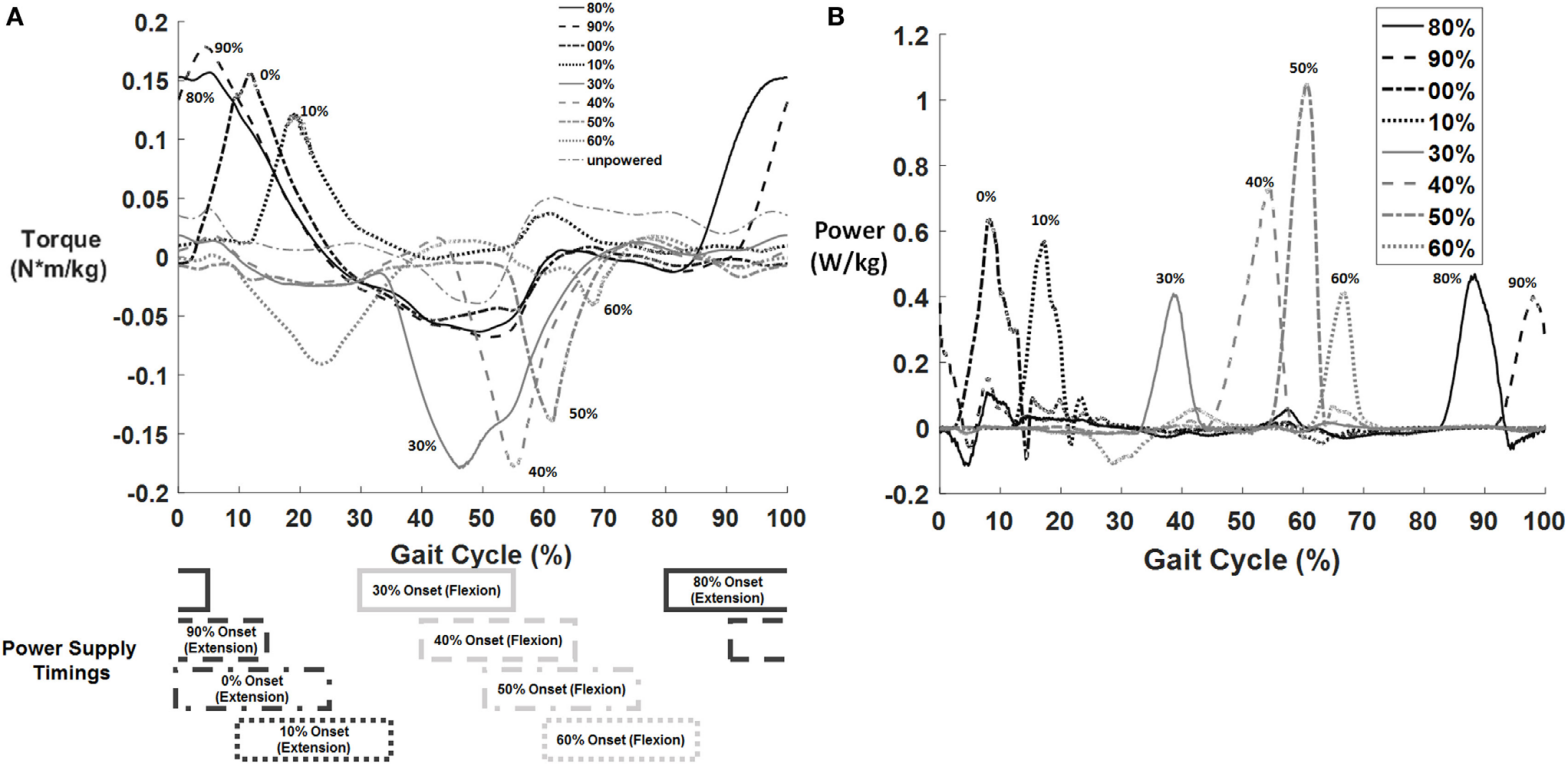
**Example of exoskeleton torque and power profiles for one subject across different timing conditions (top)**. Curves for torque **(A)** and power **(B)** are shown for only one subject (averaged across left and right side) normalized to body weight, but similar torque and power curves were observed across all subjects. The torque curve of the unpowered exoskeleton was subtracted from each of the powered curves such that the displayed graph shows only the difference between the powered and unpowered conditions. The timing for each condition is shown below the torque graph (bottom). Darker conditions correspond to hip extension and lighter to hip flexion. The duration of the power signal was always set to 25% of the gait cycle, with only the onset and offset timings varying between conditions.

The exoskeleton controller successfully produced a torque profile on the user during the activation period in each condition (Figure 4A). The peak exoskeleton torque for each condition tended to occur approximately 10–15% of the gait cycle after the onset signal. Even though the supplied pressure and duration of the *on* signal were the same across conditions, the magnitude of the exoskeleton torque tended to be larger for timings that occurred earlier in the gait cycle for both hip flexion and extension assistance. This was due to the velocity of hip movement during the activation timing as later conditions tended to have higher hip velocity in the direction of assistance lowering the overall torque (but keeping supplied power nearly constant). The hip power curves (Figure 4B) show fairly similar applied powers across conditions. Notably, the 60% flexion assist condition had both a low applied torque and a passive torque that developed during midstance; this may have negatively affected the condition. However, it is likely that the positive benefit of the swing flexion assistance overcame the potentially harmful effects of the negative power during the middle of stance as on average there was still a metabolic reduction from the unpowered condition.

### Human Biomechanics Results

**Figure 5:**
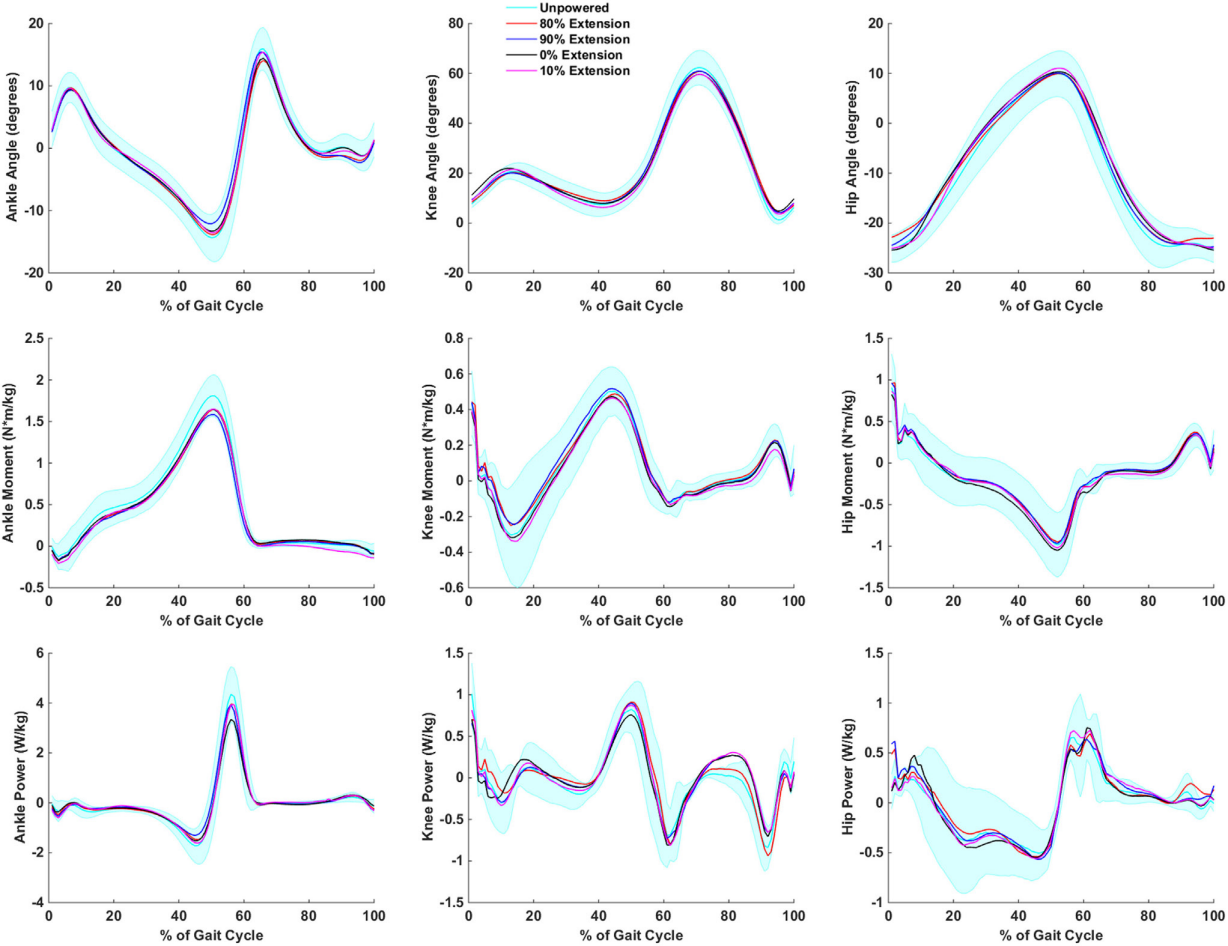
**Biomechanics of hip, knee, and ankle during powered hip extension conditions**. Joint extension is always positive, and joint flexion is negative. It is important to note that the joint torques and powers presented are a combination of exoskeleton and human joint torque and power. The first column corresponds to the ankle, the second to the knee, and the third to the hip. The first row is joint angles, the second row is joint moments, and the third row is joint powers. Data are normalized to the gait cycle (0% indicates heel strike). Data were averaged across subjects, and shaded regions represent ±1 SD for the unpowered condition only.

The largest trend in the inverse dynamics for the powered extension conditions (Figure 5) was a decline in ankle torque and power generated at the ankle relative to the unpowered condition. Depending on condition, the decline in ankle power was between 9 and 24%, while ankle torque was between 7 and 13% (Table 2). This decrease indicated a reduction in the need to generate forward power at the ankle. The kinematics at the ankle were relatively unchanged across powered extension conditions. Smaller positive hip power changes were observed indicating that earlier extension assistance reduced hip power at toe off, while later extension assistance reduced power at heel contact. No noticeable effects at the knee joint were noted.

**Table 2:** **Ankle peak moments and peak powers across conditions**.

Condition	Peak moment [(N·m)/kg]	Peak power (W/kg)
Unpowered	1.81 (0.25)	4.35 (1.10)
80% extension	1.65 (0.27)	3.96 (1.11)
90% extension	1.58 (0.52)	3.94 (1.83)
0% extension	1.64 (0.25)	3.34 (1.29)
10% extension	1.65 (0.35)	3.92 (1.26)
30% flexion	1.73 (0.33)	4.54 (1.01)
40% flexion	1.61 (0.27)	3.68 (1.11)
50% flexion	1.71 (0.22)	3.36 (0.63)
60% flexion	1.56 (0.26)	3.83 (0.87)

*Data are represented as mean (1 SD)*.

**Figure 6:**
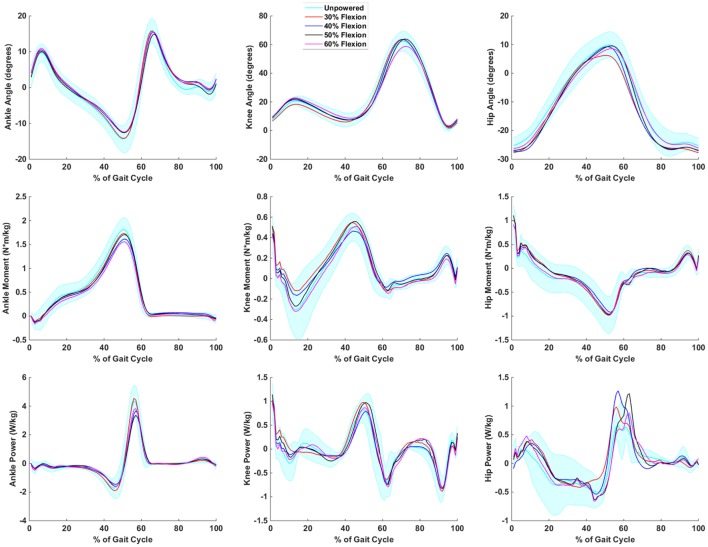
**Biomechanics of hip, knee, and ankle during powered hip flexion conditions**. Joint extension is always positive, and joint flexion is negative. It is important to note that the joint torques and powers presented are a combination of exoskeleton and human joint torque and power. The first column corresponds to the ankle, the second to the knee, and the third to the hip. The first row is joint angles, the second row is joint moments, and the third row is joint powers. Data are normalized to the gait cycle (0% indicates heel strike). Data were averaged across subjects, and shaded regions represent ±1 SD for the unpowered condition only.

The inverse dynamics for the powered flexion conditions (Figure 6) indicated a few trends at the hip and ankle but no substantial deviations from the unpowered condition at the knee. The hip angle excursion into extension was reduced in the 30% flexion condition from 10° of extension to 7°. This may have caused the need for additional positive power generation during this part of the gait cycle. This was observed at the ankle as peak positive power was the same between the unpowered and 30% flexion assist condition. However, peak positive power decreased by 13–24% across the other powered flexion conditions. Similarly, peak ankle torque decreased by 2–24% across the powered conditions (the 2% decrease corresponded to the 30% flexion condition). There were increases in peak positive hip power generation between 23 and 45% compared to unpowered in the flexion assist conditions. Thus, the biomechanics indicate that the 30% flexion condition may have had slightly unfavorable changes in joint biomechanics at the hip and ankle.

## Discussion

The purpose of this study was to determine the best timing for supplying hip power through an exoskeleton to reduce the metabolic cost of walking. The study results indicate that the optimal timing is subject specific; there is no single assistance timing that maximally reduced metabolic cost for all subjects (Table 1). However, global parameters for choosing the timing may be useful across subjects as 60–85% of the metabolic gains can be achieved simply by selecting the point that is on average best across subjects. To achieve the absolute optimal gains, practitioners likely need to tune the power activation onset on a subject-by-subject basis. In practice, tuning an exoskeleton is not an easy task, and user preference is often used to guide the tuning process. We tested the hypothesis that user preference would correspond with conditions that incurred a lower metabolic cost. We found this not to be the case, as user preference did not correspond to metabolic cost (Figure 3) within a given activation direction (flexion or extension). Subjects were either unable to sense their metabolic cost or preferred conditions based on other factors such as their stability or comfort level with a given controller. Thus, we conclude that user preference may not be an effective method to tune exoskeletons for optimal reduction of metabolic cost of walking. New paradigms are emerging in the field that use human-in-the-loop optimization to quickly find the optimal parameter set based on metabolic cost; these strategies may be promising for helping to solve this problem (Felt et al., 2014). However, this still requires the use of a metabolic analysis unit, which is not ideal. A simpler solution may be to use the assistance timing that on average reduces metabolic cost maximally across a wide range of individuals, which was the primary goal of this study.

The metabolic cost averaged across all subjects versus the actuation onset timing demonstrated a U-shaped pattern for both hip flexion and hip extension. A similar U-shaped pattern was found in a related experiment conducted with an ankle exoskeleton to determine the optimal timing for supplying plantarflexor torque (Malcolm et al., 2013). Our metabolic reduction values were consistent with other recent exoskeleton experiments where metabolic reduction was between 5 and 14% (Ronsse et al., 2011; Ding et al., 2016a,b; Seo et al., 2016); we found optimized reduction to be 9.7% for hip flexion and 10.3% for extension with respect to unpowered. It is difficult to compare directly to previous experiments as metabolic cost reduction is likely a function of both power magnitude and timing (among other variables such as subject population differences, exoskeleton structure, and variable control architecture). Thus, the optimal may vary somewhat from one system to another. However, previous hip assistance studies tend to provide peak extension torque well after heel contact such as in the study by Seo et al. (2016) (~15%) and Ding et al. (2016a) (~20%), and oscillator-based control is even later (Giovacchini et al., 2014) (~25%). For example, two recent studies in the field that used a combination of hip flexion and extension assistance to successfully reduce the metabolic cost of walking with an exoskeleton applied hip extension assistance to have a peak extension torque at ~20% of the gait cycle (Panizzolo et al., 2016) and 18% (Seo et al., 2016). Timings selected in these studies for hip extension corresponded closely with our 10% extension onset condition that applied a peak torque at 20% of the gait cycle. We found a metabolic reduction (3.8% compared to unpowered) at this condition, but our study indicates that an earlier time point might be of greater benefit; for example, the 90% extension onset condition had a peak torque at 5% of the gait cycle and reduced metabolic cost by 8.4%. In comparing the flexion assistance, these two previous studies provided peak flexion torque at ~61% (Panizzolo et al., 2016) and ~65% (Seo et al., 2016). These timings for hip flexion assistance corresponded closely with our 50% flexion onset condition, which had a peak torque at 61% of the gait cycle. This condition reduced metabolic cost by 6.1% compared to unpowered and was nearly equivalent with the 40% flexion onset condition. Our study supported this timing for hip flexion assistance with a maximum reduction either at or before the timing used in the study by Panizzolo et al. (2016) and Seo et al. (2016). Although it was beyond the scope of this study, it is possible that a combination of both hip flexion and hip extension assistance at the proper assistance timing would help to maximize the metabolic reduction further (similar to what was done in the previous studies).

Previous experiments have calculated the apparent efficiency for exoskeleton assistance (Sawicki and Ferris, 2008). Apparent efficiency is the ratio between the average exoskeleton positive mechanical power and the change in net metabolic power. Apparent efficiency changes depending on the joint and onset timing of assistance. Our earlier studies found an apparent efficiency of 0.61 for ankle exoskeleton assistance but postulated a lower value for the assistance of proximal musculature, such as the hip. We found an apparent hip efficiency ranging from 0.2 to 0.4 depending on condition using our hip exoskeleton, which agrees with the earlier predictions that hip assistance would have an apparent efficiency of 0.25–0.30 (Sawicki et al., 2009).

The biomechanical analysis helped to supplement the metabolic analysis by analyzing the changes in joint torques and powers for different conditions. The hip extension conditions may have aided gait biomechanics by reducing the peak torque and power requirements at the ankle joint. The timing of hip extension produced mixed results at the hip as earlier assistance appeared to be favorable for reducing power at toe off, but later assistance appeared to be favorable for reducing power at heel contact. During the flexion assistance conditions, the biomechanics were less favorable for early onset (30% of the gait cycle) as both hip and ankle positive power production was higher. Hip and ankle power appeared to be reduced with later powered flexion onset timings.

A primary limitation of this study was that the pneumatic actuators were tethered to a compressed air supply that is not portable. This limited the experimental protocol to treadmill walking. It would be valuable to test over ground walking in the future to ensure generalization of the results. Although it is possible to design exoskeletons with portable pneumatic systems (Shorter et al., 2011), it is not easy and greatly limits the amount of assistance. We did not want to limit the assistance to such small torque levels as it is likely that significant assistance is needed to yield substantial reductions in metabolic cost. Portable exoskeletons often use electromechanical motors (Zeilig et al., 2012; Kolakowsky-Hayner et al., 2013; Mooney et al., 2014; Buesing et al., 2015) with high gear ratios to achieve adequate torque levels similar to those achieved in this experiment (Figure 4). Another limitation of this study was that only a single walking speed (1.15 m/s) was tested during steady-state level walking. In real life, humans constantly adjust their walking speed, transition between different gait activities such as walking and standing, and do not solely locomote on level surfaces. Further testing under these scenarios is needed to fully determine the best power actuation timing.

In our study, we used a simple on/off controller that provided the same amount of power input across conditions. However, the actual torque generated (Figure 4A) is a function not only of the pneumatic cylinders but also of the exoskeleton dynamics, the interface dynamics, and the human dynamics. A pneumatic system cannot instantaneously apply torque (such as an electric motor would) but rather must build up torque due to pressurization. Peak torque tended to occur around 10% of the gait cycle after the initiation point; however, torque started to apply immediately to the user as soon as the onset point occurred. Torques that occurred outside the delivery window were due to passive user/exoskeleton interaction forces. We did not tune the controller in any way or control for delivered torque, but simply provided the same “burst” from the pneumatic system across all nine conditions. The torque actually delivered depended entirely on the dynamics of the overall system and is unsurprisingly quite different across the experimental conditions. The output power of the exoskeleton (Figure 4B) depends on factors such as soft tissue compression, human movement dynamics, and exoskeleton movement (play in the device) and thus is not exactly the same across conditions. However, the output power (as displayed in Figure 4B) was fairly consistent across the different conditions. The peak power was not always the same in each condition, but the total energy (area under the curve) was similar in each condition. It is possible that different results would be found if the exoskeleton controlled for specific torque levels rather than specific power input levels. These results are primarily applicable for pneumatic systems (or hydraulic systems) due to the dynamics of the system, but may have use in electromechanical systems. Further experiments on electromechanical systems would be necessary to verify these conclusions.

Numerous industry and research groups are designing exoskeletons that include actuated hip joints (Arazpour et al., 2014; Farris et al., 2014; Giovacchini et al., 2014; Asbeck et al., 2015). The findings of this study are useful to help designers to properly time exoskeleton assistance to reduce the metabolic cost of walking. This includes applications for military use, industrial grade exoskeletons, and medical exoskeletons for disabled or elderly individuals. Studies on the effects on biomechanics and metabolic cost of human subjects wearing exoskeletons are necessary to help progress the field (Ferris, 2009). Numerous market predictions are forecasting that the exoskeleton market will grow exponentially within the next 5 years from current market of $16.5–$2.1 billion (Austin, 2015). Exoskeleton controllers and hardware will need significant advancement to achieve this ambitious goal, and human subject studies with exoskeletons such as this one will provide a critical and necessary element in validating the utility of exoskeleton technology.

## Ethics Statement

The study protocol was approved by the University of Michigan Institutional Review Board. Subjects read an IRB approved consent form and were additionally verbally informed as to the experimental procedure and expectations. They gave written informed consent to participate in the study.

## Author Contributions

AY helped in conceiving the study concept and design, acquiring the data, analyzing and interpreting the data, and drafting the manuscript. JF and HG helped in acquiring the data, analyzing and interpreting the data, and drafting the manuscript. DF helped in conceiving the study concept and design, drafting and revising the manuscript, obtaining funding, and supervising the study.

## Conflict of Interest Statement

The authors declare that the research was conducted in the absence of any commercial or financial relationships that could be construed as a potential conflict of interest.
